# Eye Drift Signal Analysis Caused by Goggle Slippage in vHIT Measurements: A Signal Processing Perspective

**DOI:** 10.3390/s26092880

**Published:** 2026-05-05

**Authors:** Ha Ngoc Khoan, Le Ky Bien, Tran Thi Nhan, Tran Van Nghia

**Affiliations:** 1 Cyclotron Centre, 108 Military Central Hospital, Hanoi 100000, Vietnam; hangockhoan.bv108@gmail.com; 2 Academy of Military Science and Technology, Hanoi 100000, Vietnam; lekybien@gmail.com; 3 Faculty of New Energy, Electric Power University, Hanoi 100000, Vietnam; nhantt@epu.edu.vn; 4 Air Force–Air Defense Technical Institute, Hanoi 100000, Vietnam

**Keywords:** eye drift, vHIT, VOR, goggle slippage, gain error, saccade, signal correction

## Abstract

This Technical Report presents a quantitative signal processing approach to analyze and correct eye drift during vestibulo-ocular reflex (VOR) measurements using the video Head Impulse Test (vHIT). The objective is to determine the extent of drift caused by goggle slippage—a technical artifact that can distort the VOR gain index. A total of 57 impulses were categorized into three protocols: Lateral, LARP, and RALP. For each impulse, peak head velocity and eye drift (estimated from the average velocity during the pre- and post-impulse rest periods) were extracted using a custom signal processing pipeline implemented in MATLAB R2020b and Python 3.11 64 bit. Results showed the highest drift in the RALP group (−7.41 deg/s) and the lowest in the LARP group (−3.08 deg/s). The correlation between head velocity and drift was most prominent in the RALP group (r > 0.7), highlighting the impact of stimulation direction on goggle stability. This study proposes a drift detection method to be integrated into VOR correction algorithms, thereby enhancing gain analysis and saccade detection in automated systems.

## 1. Introduction

The Vestibulo-Ocular Reflex (VOR) is one of the most critical physiological reflex mechanisms in humans. It enables the stabilization of retinal images during head movements. When the head rotates, the vestibular system in the inner ear detects this motion and instantly transmits signals to the extraocular muscles, directing the eyes to move in the opposite direction to maintain a fixed gaze. This reflex operates at very high speed, with latencies typically under 10 ms [1], and is essential for maintaining clear vision in daily activities.

(One of the most) modern and effective methods for evaluating VOR function is the video Head Impulse Test (vHIT). This method utilizes goggles integrated with inertial measurement units (IMUs) and high-speed cameras to accurately capture head and eye velocities during rapid and unexpected head impulses in predetermined conditions directions [2,3,4]. From the acquired data, quantitative metrics such as VOR gain, presence of overt and covert saccades, and other ocular motor characteristics can be analyzed to assess the function of the semicircular canals [2,3,4]. However, the accuracy of measurements obtained from the vHIT is highly dependent on the spatial positioning of the device. When the goggles experience slight slippage, often due to high-velocity impulses or difficult-to-maintain stimulus directions, the recorded eye velocity signals can become distorted. According to Yip et al. (2016), although the video-based method (vHIT) offers higher sensitivity, it is more prone to mechanical errors compared to manual examination techniques, potentially resulting in artificial signals that do not accurately reflect physiological eye velocity [4,5,6].

Several studies have acknowledged the presence of eye drift and its association with measurement quality. Suh [1] indicated that goggle slippage reduces measurement accuracy and increases error rates in VOR assessments. Research by G. T. Mantokoudis et al., [6] and Halmagyi et al. [3] also found that improper goggle fitting could lead to significant measurement errors, particularly during high-velocity head impulses. However, most of these studies have primarily described the phenomenon qualitatively, lacking systematic quantitative analyses of drift magnitude across different test protocols, and without clarifying the quantitative relationship between drift and head velocity, a key factor potentially responsible for critical measurement errors.

This paper proposes a quantitative approach to assess eye drift in three vHIT protocols: Lateral, LARP, and RALP. Based on 57 impulses recorded using the ICS Impulse system, the study calculates average eye drift and peak head velocity, and examines their linear correlation within each group. The findings reveal protocol-dependent differences in drift magnitude and confirm the role of head velocity in amplifying drift, thereby identifying a technical source of error in vHIT measurements. Building on these results, this study provides a technical proof-of-concept for a drift correction algorithm, emphasizing mathematical robustness and optimization despite the limited dataset, and lays the foundation for future applications in VOR modeling and automated saccade detection algorithms with compensation for technical artifacts.

## 2. Methodology

### 2.1. Data and Equipment

The study utilized 57 head impulse recordings acquired using the ICS Impulse device (Natus Medical Denmark ApS, Taastrup, Denmark) at a sampling frequency of 250 Hz from a healthy volunteer. Prior to data collection, the system was calibrated according to the standard manufacturer’s routine to ensure the accuracy of head and eye velocity measurements The study protocol was approved by the Institutional Review Board in Biomedical Research of the 108 Military Central Hospital (registered under code IRB-VN01.108; Approval No. 2703/QD-BV), and the participant provided written informed consent prior to data collection. The recordings were categorized into three vHIT test protocols: Lateral (VOR125–VOR148), LARP (VOR149–VOR164), and RALP (VOR165–VOR181). Each data file contained three columns of discrete-time signals: time (s), head velocity (deg/s), and eye velocity (deg/s). The data were stored in .txt format and preprocessed using MATLAB 2020b to extract 57 impulses into 57 individual data files.

In addition to the 57 impulses analyzed in this study, a separate dataset of approximately 700 head impulse recordings was used exclusively for parameter optimization in the proposed correction model. These recordings were obtained from routine measurements using the same ICS Impulse system under similar acquisition conditions.

This optimization dataset is independent of the 57-impulse dataset used for statistical analysis. No overlap exists between the two datasets.

### 2.2. Signal Preprocessing

The signal processing procedure follows a structured six-step pipeline. The input to the pipeline consists of three synchronized discrete-time arrays per recording: eye velocity $v_{e}$, head velocity $v_{h}$, and time $t_{d}$, along with the total number of impulse files $N_{file}$.

Step 1—Trimming and windowing. Raw signals are trimmed to remove edge noise, and a time window from 0.2 s to 0.7 s is extracted around each head impulse. This window consistently captures the onset, peak, and recovery phases of the vestibulo-ocular response.

Step 2—Identification of rest regions. A binary mask array mask(i,j) is constructed to label the non-movement (resting) phases of each recording, defined as time samples satisfying $t_{d}<0.05$ s or $t_{d}>0.5$ s (Equation (1)). These regions serve as the reference baseline for drift estimation.

Step 3—Computation of drift values. Using the masked rest regions, three drift quantities are computed: eye velocity drift d_e (Equation (2)), head velocity drift d_h (Equation (3)), gaze error e_gaze (Equation (4)).

For clarity, v_e(t) and v_h(t) denote the eye and head velocity signals, respectively, as functions of time. The gaze error is defined as:

e_{gaze}(t) = v_e(t) + v_h(t)(1)

The nonlinear function g(·) represents the correction applied to the eye velocity signal.

e_gaze_(i,j) = g(v_e_(i,j),θ) + v_h_(i,j) (2)

and gaze error drift $d_{gaze}$ (Equation (5)). These values represent the slow, non-physiological offset present in each signal during stationary phases.

Step 4—Variance and standard deviation. For each impulse, the variance and standard deviation are computed for eye velocity (${\sigma_{e}}^{2}$, $\sigma_{e}$, Equations (3) and (4)), head velocity (${\sigma_{h}}^{2}$, $\sigma_{h}$, Equations (5) and (6)), and gaze error (${\sigma_{gaze}}^{2}$, $\sigma_{gaze}$, Equations (6) and (7)). These statistics quantify the degree of signal instability and are used in subsequent correlation analysis.

Step 5—Peak head velocity extraction. The peak head velocity $v_{max}$ is determined as the maximum absolute value of $v_{h}$ across all samples within each impulse window (Equation (8)). This scalar metric represents the mechanical stimulus intensity delivered to the vestibular system.

Step 6—Grouping and classification. A summary table is constructed by aggregating the computed statistics across all 57 impulses. Files are then categorized into three groups—Lateral, LARP, and RALP—based on their stimulation direction. Signal stability within each group is assessed by comparing drift magnitude and standard deviation, forming the basis for the correlation analysis presented in Section 3.

### 2.3. Grouping and Characterization

The data were divided into three groups based on the semicircular canal stimulation direction: Lateral (horizontal), LARP (left anterior–right posterior), and RALP (right anterior–left posterior). Each group was processed independently to calculate the following metrics: (1) average eye drift, (2) head drift and peak head velocity, (3) Pearson correlation between statistical variables to identify those with a linear influence on gaze error.

### 2.4. Statistical Analysis and Processing Tools

Data preprocessing (file parsing, impulse segmentation) was performed in MATLAB 2020b (MathWorks, Natick, MA, USA). Statistical analysis, correlation computation, and figure generation were carried out in Python 3.x using the pandas, matplotlib, and seaborn libraries. The gradient descent optimization was implemented in MATLAB 2020b.

### 2.5. Proposed Correction Method

Based on the results above, we observed that the signals frequently exhibit nonlinear drift caused by system noise, accumulated filter errors, or sensor drift over time. This type of nonlinear error affects the entire impulse signal captured by the sensor and has a significant impact during the head’s stationary phase.

To eliminate this slow-drift component while preserving the true dynamic characteristics of the signal, we propose a correction method based on a nonlinear regression function. This method consists of three main steps: (1) Evaluate Kendall’s rank correlation between statistical features of the head velocity signal, eye velocity signal, gaze error, and eye drift; (2) Construct a nonlinear function and determine its coefficients such that the standard deviation of the gaze error is minimized; (3) Assess the effectiveness of the nonlinear function in reducing the standard deviation of the gaze error when applied to the eye velocity signal.

This technique enables the removal of smooth drift components without affecting the fast fluctuations that characterize eye movements. Kendall’s Tau-b is a rank correlation coefficient used to measure the ordinal association between two variables. It is especially useful when working with ranked data, as opposed to Pearson’s correlation, which assumes numerical values. By analysing Kendall’s Tau-b values, one can identify dependencies among statistical features to inform the design of the nonlinear correction function for the eye velocity signal. The Kendall’s Tau-b correlation results from two square matrices, with both rows and columns equal to the number of variables under consideration. The output consists of two parameters: the correlation coefficient ($\tau_{b}$) and the corresponding statistical significance value ($\rho_{b}$).

The correlation coefficient ($\tau_{b}$) is a real number ranging from −1 to 1. Its interpretation is as follows:

- Values close to −1 indicate a strong negative correlation between the variables at the respective row and column positions of the matrix.
- Values near 0 indicate no correlation between the variables.
- Values close to 1 indicate a strong positive correlation between the statistical variables.

The significance value ($\rho_{b}$) is a real number in the range [0, 1], interpreted as follows:

- If the value is less than or equal to 0.05, the two variables are statistically correlated.
- If the value is greater than 0.05, the variables are either not statistically correlated, or the sample size may be insufficient to reveal a statistical relationship.

**Figure 1:**
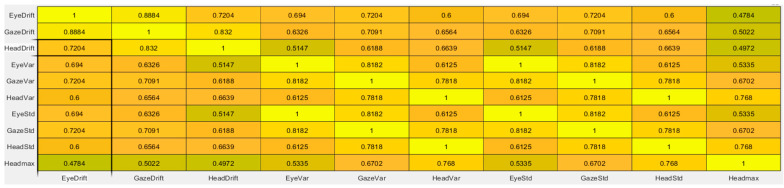
Correlation matrix between kinematic variables.

**Figure 2:**
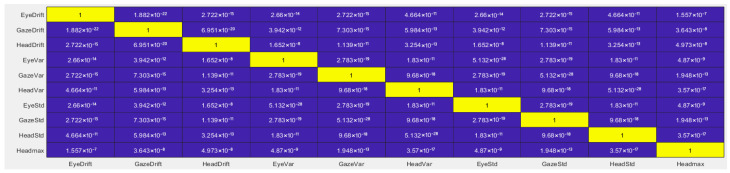
Statistical significance values between statistical variables.

The computation of $\rho_{b}$ and $\tau_{b}$ values was performed using the corr (X) function in MATLAB, where the input X is a two-dimensional matrix containing the variables to be correlated, arranged by columns. Figure 1 presents the resulting $\tau_{b}$ correlation matrix, while Figure 2 shows the corresponding $\rho_{b}$ significance matrix for the set of statistical variables:

(3) $$X=[d_{e},\;d_{h},\;e_{gaze},\;d_{gaze},\;{\sigma_{e}}^{2},\;\sigma_{e},\;{\sigma_{h}}^{2},\;\sigma_{h},\;{\sigma_{gaze}}^{2},\;\sigma_{gaze},\;v_{max}]$$

The objective of the nonlinear function is to eliminate the influence of nonlinear errors caused by goggle slippage on the eye movement signal. In other words, the standard deviation of the gaze error should be minimized as much as possible. Therefore, a nonlinear function must be constructed based on statistical variables whose $\rho_{b}$ and $\tau_{b}$ values indicate the strongest statistical correlation with $\sigma_{gaze}$. The authors observed that the standard deviations of head velocity $\sigma_{h}$ and eye velocity $\sigma_{e}$ exhibited the highest $\rho_{b}$ and $\tau_{b}$ correlation values with the standard deviation of the gaze error. Therefore, the proposed nonlinear function is formulated as a second-order polynomial applied to the eye velocity vector.

Let the second-order nonlinear function applied to the eye velocity ($v_{e}$) be defined as:

(4) $$g\left(v_{e}(i,j),\theta \right)=\theta_{1}.v_{e}^{2}\left(i,j\right)+\theta_{2}.v_{e}(i,j)+\theta_{3}$$

The drift of the gaze error is calculated as defined in Equation (5):

(5) $$d_{gaze}\left(i\right)=\frac{1}{\sum_{j}mask\left(i,j\right)}\sum_{j}e_{gaze}\left(i,j\right).mask\left(i,j\right)$$

The standard deviation of the gaze error is calculated as defined in Equation (7)

(6) $$\sigma_{gaze}(i)=\sqrt[{2}]{\frac{1}{\sum_{j}mask\left(i,j\right)}\sum_{j}^{N_{file}}{\left(e_{gaze}\left(i,j\right).mask\left(i,j\right)-d_{gaze}\left(i\right)\right)}^{2}}$$

To optimize the coefficients for minimizing the value of $\sigma_{gaze}$, the authors applied a nonlinear optimization method using gradient descent, The optimization was performed using the independent 700-impulse dataset described in Section 2.1 including Lateral, RALP, and LARP impulses. The optimization was performed over the parameter vector $\theta =[\theta_{1},\theta_{2},\theta_{3}]$.

Parameter initialization: The optimization was initialized with θ(0) = [0, 1, 0], representing a linear pass-through function with no quadratic correction term. The learning rate was set to α = 0.7, selected empirically within the range [0.6, 0.8] based on convergence stability. A second-order polynomial was chosen as the correction model because Kendall analysis identified σh and σe as the dominant correlates of σ_gaze_, and a quadratic form provides sufficient flexibility to model velocity-dependent nonlinear drift without risk of overfitting given the smooth, low-frequency nature of the drift component. Convergence was defined as ||θ(n + 1) − θ(n)||_2_ < 10^−4^, or a maximum of 700 iterations, whichever was reached first. Overfitting was mitigated by the low dimensionality of the model (3 parameters) relative to the dataset size (700 samples).

The core idea of the algorithm is to iteratively adjust the parameter values in the direction opposite to the gradient of the partial derivative of $\sigma_{gaze}$, evaluated at each step θ(n), until the RMS of $\sigma_{gaze}$ reaches a near-zero minimum. The gradient $\bar{\nabla J(\theta )}$ represents the average gradient of the gaze error standard deviation, and is computed as follows:

(7) $$\bar{\nabla J(\theta )}=\frac{1}{N}\sum_{i=0}^{N-1}\nabla J(\theta_{i})$$

Here, $\nabla J\left(\theta_{i}\right)$ represents the RMS value of the error vector $\sigma \left[z\right]$ evaluated at a specific value of $\theta_{i}$ within a predefined range.

In essence, gradient-based optimization involves iteratively computing the partial derivatives of the cost function with respect to each parameter $\theta_{i}$ over a set of known values. After each iteration, the parameter vector $\theta^{*}$ is updated according to the following rule:

(8) $$\theta^{*}=\theta -\alpha \cdot \nabla J(\theta )$$

The coefficient α is referred to as the learning rate, and it is typically selected within the range of 0.6 to 0.8 to balance training quality and convergence speed. The iterative process is terminated either when a convergence condition is met or when the number of iterations reaches the predefined limit set at the beginning of the optimization.

Upon completion of the optimization algorithm, the optimal parameter set $\theta^{*}$ for the nonlinear function model is obtained. In this study, the algorithm converged after 700 iterations on a dataset of 700 head impulses, resulting in the following optimal parameters: $\theta^{*}=[-81.06;\;91.16;\;411.89]$.

## 3. Results

Analysis of data from three Head Impulse Test (HIT) groups, comprising a total of 57 impulses, revealed notable trends in eye velocity drift and its relationship with peak head velocity. The results are presented for each protocol: Lateral, LARP, and RALP. In addition, correlations between eye drift and other statistical variables were evaluated to identify those with linear relationships, serving as a basis for nonlinear regression optimization across the combined signal sample space of all three impulse groups.

### 3.1. Lateral Group (VOR125–VOR148)

**Figure 3:**
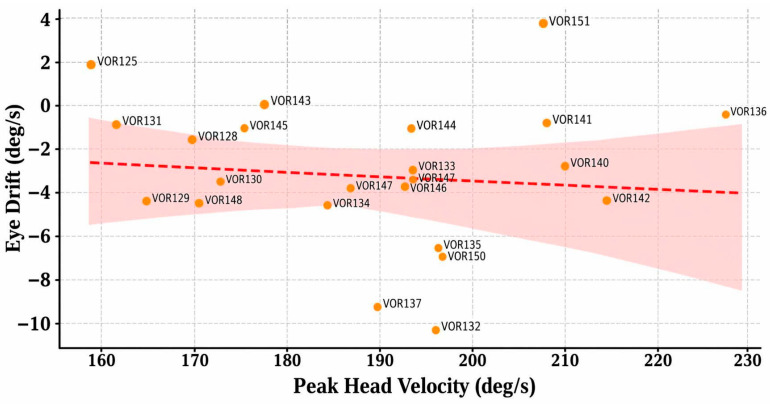
Correlation between peak head velocity and eye drift in the Lateral vHIT group (VOR125–148).

**Figure 4:**
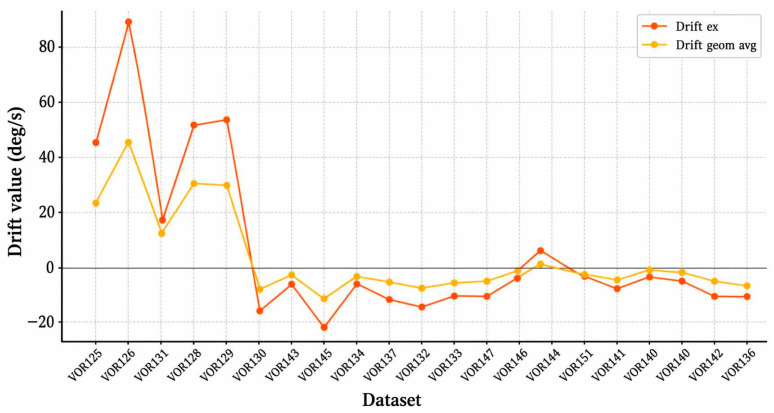
Distribution and variation trend of eye drift in vHIT recordings.

This group included 24 impulses. The average eye drift was −3.23 deg/s, with an average head velocity of 189.28 deg/s. Figure 3 shows a positive correlation trend between head velocity and eye drift, particularly for impulses with velocities exceeding 200 deg/s. This indicates that goggle slippage is more likely to occur during high-intensity lateral stimulation, see Figure 4.

The detailed statistical parameters for these correlations, including the coefficient of determination (R^2^) and significance levels (*p*), are summarized in Table 1.

### 3.2. LARP Group (VOR149–VOR164)

**Figure 5:**
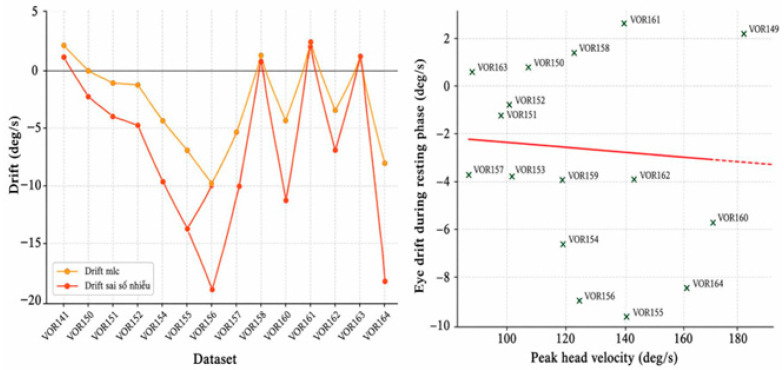
Eye drift trend and correlation between head velocity and eye drift (LARP group).

The LARP group consisted of 16 impulses, with an average eye drift of −3.08 deg/s and the lowest average head velocity among the three groups (~127.63 deg/s). Although the LARP group showed a statistically significant correlation (r = 0.72, *p* < 0.001), the scatter distribution did not exhibit a clear monotonic trend compared to the Lateral and RALP groups. Therefore, the practical strength of this relationship may be less pronounced. This may reflect more stable measurement conditions in this dataset when testing along the LARP direction, see Figure 5.

### 3.3. RALP Group (VOR165–VOR181)

**Figure 6:**
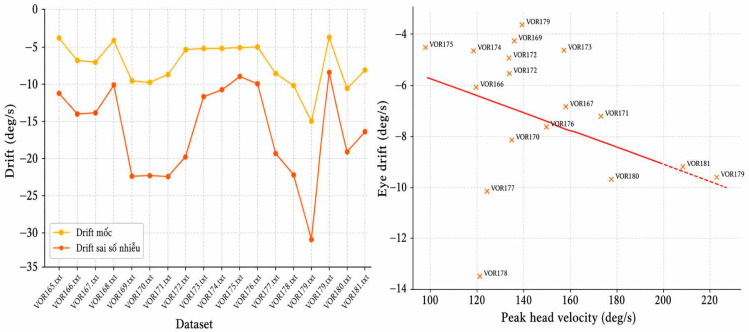
Eye drift trend and correlation between head velocity and eye drift (RALP group).

The RALP group included 17 impulses, with an average eye drift of −7.41 deg/s and an average head velocity of 153.09 deg/s. This group exhibited the highest eye drift and the greatest variability. Figure 6 shows a clear correlation between head velocity and eye drift, particularly for impulses VOR178–181. This suggests that the RALP direction is maybe more susceptible to measurement errors caused by goggle slippage, see Table 2.

**Figure 7:**
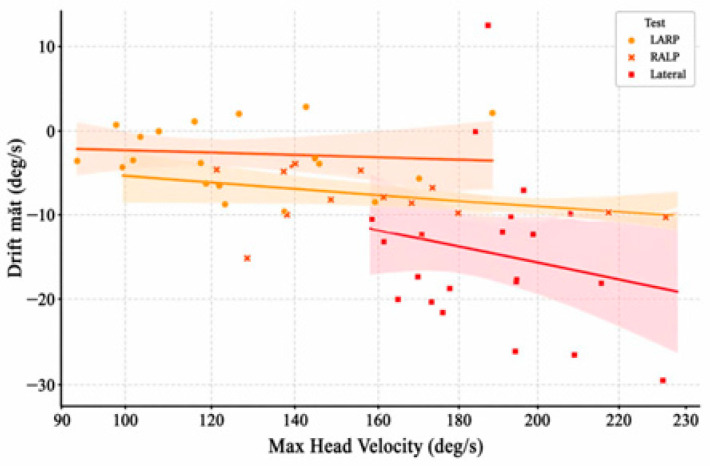
Comparison of correlation patterns by test type.

Figure illustrates the change in $\sigma_{gaze}$ before and after applying the nonlinear function with the optimized coefficients to the eye velocity signal. It is evident that the standard deviation of the gaze error is significantly reduced when the nonlinear function is applied to the eye movement signal. After applying the optimized nonlinear correction, $\sigma_{gaze}$ approaches approximately zero, see Figure 7.

**Figure 8:**
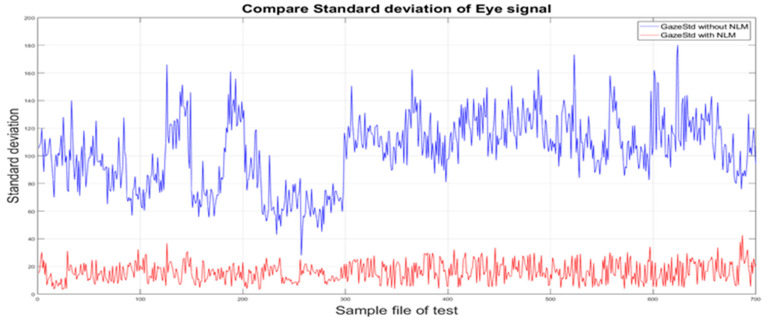
Comparison of gaze error standard deviation before and after nonlinear correction.

The results obtained from the proposed correction algorithm indicate a substantial reduction in drift-related artifacts during resting phases. At the same time, the dynamic characteristics of eye movements during high-velocity phases appear to be preserved, see Figure 8.

However, it should be noted that this assessment is based on internal signal consistency metrics rather than external physiological ground-truth validation. Therefore, while the correction method shows potential for improving signal quality and reducing false saccade detection, further validation is required to confirm its physiological accuracy.

To assess whether drift magnitude differed significantly across stimulation directions, a Kruskal–Wallis test was applied, yielding H = 13.49, *p* = 0.0012. Post hoc pairwise comparisons using Mann–Whitney U tests with Bonferroni correction (α = 0.0167) indicated that the RALP group exhibited significantly higher drift than both the Lateral group (*p* = 0.0003) and the LARP group (*p* = 0.015), whereas no significant difference was observed between Lateral and LARP (*p* = 0.431).

## 4. Discussion

The findings of this study indicate that eye velocity drift occurs across all three Head Impulse Test (HIT) protocols—Lateral, LARP, and RALP—with varying magnitudes and patterns. Among these, the RALP group exhibited the highest average drift (−7.41 deg/s), whereas the LARP group showed comparatively lower drift values.

These observations suggest that the RALP condition may be associated with increased measurement variability. However, given the single-subject design, this finding should be interpreted cautiously and not as an inherent property of the stimulation direction.

The correlation analysis shows that, in the Lateral and RALP groups, eye drift tends to increase with head velocity. This relationship may reflect the influence of inertial forces and the mechanical stability of the wearable system under stronger stimulation. In contrast, the LARP group, which exhibited lower head velocities and smaller drift variability, appears to reflect more stable measurement conditions.

The application of drift correction improved signal consistency in this single-subject dataset. The average eye velocity and standard deviation after correction showed reduced slopes and narrower fluctuations. These changes suggest a reduction in non-physiological drift components after correction, although the extent of this reduction requires further validation.

These findings are consistent with previous studies. Suh et al. [1] and Mantokoudis et al. [6] reported that vHIT measurement errors largely stem from technical issues such as goggle slippage or improper setup. Halmagyi et al. [3] and Rey-Martinez et al. [7] also noted that deviations in gain and saccade detection may reflect measurement artifacts rather than true vestibular dysfunction. Anson et al. [8] and Wong et al. [9] emphasized that small saccades are particularly susceptible to misidentification in the presence of drift. Van Dooren et al. [2] and Striteska et al. [10] further highlighted the influence of viewing distance and device configuration on signal stability. More recent work by Xu et al. [11] and Hunter & Cullen [12] underscores the importance of standardized acquisition protocols in reducing technical variability.

From a technical perspective, quantifying and correcting drift can improve signal quality and may serve as an indicator of recording stability. In applications involving VOR modeling or covert saccade detection, uncorrected drift may introduce substantial errors, particularly when saccades occur near resting phases. Therefore, drift correction may be considered a useful preprocessing step, especially in automated analysis systems.

Despite its technical contributions, this study has several limitations. The primary limitation is the small sample size (N = 1), which defines this work as a technical proof-of-concept rather than a clinical study. Consequently, the protocol-specific observations (e.g., higher drift in RALP) should be interpreted as preliminary.

Additionally, although the optimization procedure utilized a separate dataset of approximately 700 impulses, clearer documentation of the data source is required to ensure reproducibility. The evaluation was performed using internal signal quality metrics (σ_gaze_) without external ground-truth validation. Therefore, the extent to which the correction preserves true physiological eye movement remains uncertain.

Future work should incorporate multi-subject datasets, repeated measurements, and external reference systems such as scleral search coils to quantitatively assess correction accuracy and generalizability.

## 5. Conclusions

This paper presented a quantitative study of eye velocity drift in the video Head Impulse Test (vHIT) through the analysis of 57 head impulses classified into three stimulation direction groups: Lateral, LARP, and RALP. The results showed that drift was present in all three groups, though with markedly different magnitudes. The RALP and Lateral groups, which exhibited higher average head velocities, also showed greater drift levels and a positive correlation between drift and head velocity. In contrast, the LARP group demonstrated more stable signals, with smaller drift and lower standard deviation.

This analysis underscores the role of technical factors particularly goggle slippage in affecting the accuracy of vHIT measurements. Such artifacts, although not indicative of pathology, may be misinterpreted as signs of vestibular dysfunction if drift is not properly managed. The study highlights the importance of detecting and quantifying drift as a crucial step to improve input signal quality, thereby enhancing the performance of automated algorithms for covert/overt saccade detection, VOR gain estimation, and reliable vestibular function assessment.

In addition, the application of a second-order nonlinear regression-based drift correction method proved effective in improving signal stability and reliability. Based on this foundation, future developments could focus on designing automatic drift filtering models integrated into real-time vHIT measurement systems. Such systems could alert users to potential anomalies during recording or apply preprocessing before post-analysis. Furthermore, future research should expand drift analysis to patients with vestibular disorders to gain a deeper understanding of the relationship between drift phenomena and the physiological function of the vestibulo-ocular system, ultimately enhancing more accurate and personalized clinical diagnoses.

## Figures and Tables

**Table 1:** Statistical summary of the relationship between peak head velocity and eye drift.

Protocol	n	Pearson (r)	95% CI for r	R^2^	*p*-Value
Lateral	19	0.68	[0.33, 0.87]	0.46	<0.01
LARP	19	0.72	[0.39, 0.88]	0.52	<0.001
RALP	19	0.75	[0.45, 0.90]	0.56	<0.001

95% CIs were computed using Fisher’s z-transformation. All three groups showed statistically significant positive correlations between peak head velocity and eye drift.

**Table 2:** Comparison of metrics across the three groups.

Group	Number of Impulses	Mean Drift (deg/s)	95% CI	Mean Head Velocity (deg/s)	Drift Standard Deviation
Lateral	24	−3.23	[−4.55, −1.91]	189.28	3.30
LARP	16	−3.08	[−5.14, −1.02]	127.63	4.21
RALP	17	−7.41	[−8.86, −5.96]	153.09	3.06

The 95% confidence intervals for mean drift were [−4.55, −1.91] deg/s (Lateral), [−5.14, −1.02] deg/s (LARP), and [−8.86, −5.96] deg/s (RALP), indicating that the RALP group is distinguishably higher in drift magnitude compared to the other two protocols.

## Data Availability

The data are not publicly available due to privacy or ethical restrictions.
